# Estimation of the economic benefits for the public health system related to salt reduction in Costa Rica

**DOI:** 10.1371/journal.pone.0279732

**Published:** 2023-02-17

**Authors:** Jaritza Vega-Solano, Karol Madriz-Morales, Adriana Blanco-Metzler, Eduardo Augusto Fernandes-Nilson

**Affiliations:** 1 Former Researcher at the IDRC Project 108167, FUNDEVI-INCIENSA Costa Rica, Costa Rican Institute of Research and Teaching in Nutrition and Health (INCIENSA), Cartago, Costa Rica; 2 Planning Department, Ministry of Health, Secretariat of National Food and Nutrition Policy, San José, Costa Rica; 3 Costa Rican Institute of Research and Teaching in Nutrition and Health (INCIENSA), Cartago, Costa Rica; 4 Centre for Epidemiological Research on Nutrition and Health, University of Sao Paulo, Sao Paulo, Brazil; The University of Mississippi Medical Center, UNITED STATES

## Abstract

Excessive salt and sodium intake are strongly associated with high blood pressure and increased risk of cardiovascular disease. High blood pressure in turn is the main risk factor for the global burden of morbidity and mortality. The prevalence of this disease in the adult population of Costa Rica in 2018 was 37.2%. Costa Rica has limited information on the economic costs for the public health system and related of the prevalence of this type of disease mediated by dietary factors such as salt intake. Objective: to estimate the economic benefits for the public health system related to salt reduction in Costa Rica for the year 2018. Methodology: estimation of the economic benefits for the public healthcare costs and productivity losses associated to reducing the per capita salt consumption of Costa Ricans to 5g/day, including the estimation of the Years of Life Productive Lost and of the direct costs on consultations, hospitalizations, and medications for the Costa Rica Social Security System. Results: The total annual costs of hospitalization, consultations, and medications attributable to excessive salt intake in the population older than 15 years of age for the year 2018, were estimated at USD $15.1 million. The highest were in hospitalizations (53%), followed by consultations and medications (32% and 15%, respectively). Conclusion: NCDs caused by excessive salt intake represent important economic losses for the country, not only in terms of direct health costs, but also indirect due to the increase in years of potential life lost due to premature deaths because of CVD, which causes significant losses of human capital and, therefore, to the economy and the development of Costa Rica.

## Introduction

Excessive salt and sodium intake are strongly associated with high blood pressure and increased risk of cardiovascular disease (CVD) [1]. High blood pressure (hypertension) in turn is the main risk factor for the global burden of morbidity and mortality and is estimated to cause about 9.4 million deaths each year; equivalent to more than half of annual deaths attributed from CVD [2].

The most recent Global Burden of Disease Study (2019) reported that a high-sodium diet as the leading dietary risk factor for deaths and disability-adjusted life years (DALYs), responsible for 44.9 million DALYs lost and 1.89 million deaths [3].

Noncommunicable diseases (NCDs) represent the main cause of death in Costa Rica. In 2018 they were responsible for 83% of all deaths and 29% corresponded to cardiovascular diseases (CVD) [4]. The prevalence of high blood pressure (or hypertension) in the population over 19 years of age in Costa Rica for that same year was 37.2% (diagnosed 32.4% and undiagnosed 4.8%) [5].

In the country, the most recent studies indicate that sodium intake (4.42 grams/person/day (g/p/d)) was more than double the maximum recommendation established by the Organization World Health Organization (WHO) (2000mg of sodium equivalent to 5 g of salt/p/d) [6, 7]. Recent modeling studies estimated that a 15 to 46% decrease in daily salt intake could prevent 5 to 13% of deaths due to CVD [4].

The WHO considers salt reduction interventions in the population as one of the most cost-effective public health measures, or "Best-buys", for reducing the global burden of NCDs [8]. However, the costs of CVD, related to NCDs from poor diets, rising obesity, and aging societies, are likely to continue to rise. Therefore, it is very important to address the burden of these diseases in terms of mortality, morbidity and costs to health systems and to societies [9].

According to the national public health institution responsible for health care provision, the Costa Rican Social Security System (CCSS for its acronym in Spanish), in 2012 the cost of hypertension care was greater than USD $ 80,000, representing 3.47% of the spending on social security [10]. Additionally, a 5% reduction of the prevalence of hypertension, considering the full implementation of the National Plan for the Reduction of Salt/Sodium Consumption in Costa Rica 2011–2021, would save over 15% of the costs of hypertension from 2022–2031 [11].

Despite the above figures, there is limited information at the national level on the prevalence and on the economic costs for the public health diseases mediated by dietary factors such as salt intake [4, 11]. This situation is consistent with the context of low- and middle-income countries where the economic costs of CVD is unknown [12].

For this reason, methodologies have been developed for modeling the impact on health and the gradual incorporation of economic analysis of the direct and indirect cost of diseases and the cost-benefit, cost-utility and cost-effectiveness of policies and interventions [13]. These economic studies may provide evidence to support CVD prevention programs, as cost-effective measures, compared to the costs associated with treating these diseases and related conditions [9].

In Costa Rica, the Preventable Risk Integrated ModEl (PRIME), a macrosimulation comparative risk assessment methodology, has already been used to estimate the health impact of reducing dietary salt at the population level [4], and represents a feasible, cost-saving, and useful tool for decision makers at the public health level. However more evidence on the direct and indirect economic costs of excessive is needed in the country [14, 15].

Estimating the costs associated with excess salt/sodium and the economic impact of interventions, based on an analysis of health policies and salt intake scenarios, can contribute to greater profitability of public policies for salt/sodium reduction [11].

The objective of this work is to estimate the economic benefits for the public health system related to salt reduction in Costa Rica for the year 2018.

## Methodology

This study provides cost-of-illness analysis, from the perspective of the Costa Rican Social Security System and productivity losses associated with premature mortality from hypertension and CVD, in 2018. We estimated the economic benefits for the public healthcare costs and productivity losses associated to reducing the per capita salt consumption of Costa Ricans to 5g/day, including the estimation of the Years of Potential Life Lost (YPLL) and of the direct costs on consultations, hospitalizations and medications for the CCSS (S1 Appendix).

### Cost-of-illness modeling of premature deaths

The estimated YLL, which are part of the estimates for DALYs, were calculated according to the methodology used by the Global Burden of Disease (GBD) studies [16]:

YLL=NxL

Where: N = the number of deaths from CVDs averted or postponed estimated through the PRIME [4, 17]; and L = the standard life expectancy at the age of death in years for the Costa Rican Population [18].

The YPLL were estimated by using the Human Capital Approach, which calculates the current value of potential time in the workforce (the measure of productivity) using country-specific data for 2018 [19]. The YPLL were calculated by multiplying the YLL from age 15 to the pension age (60 years for women, and 65 years for men) by the average national wage and the labor force participation estimates from Costa Rican references, by The National Institute of Statics and Censes (INEC, for its acronym in Spanish) [20].

### Direct costs of disease to the Costa Rican health system

The attributable costs to excessive sodium consumption used previously, validated cost-of-illness model [12]. It is based on the findings of the meta-analysis of randomized controlled trials, which established the age-specific relationship between salt and blood pressure [21], and other meta-analysis that link hypertension with CDV [22].

In this model, salt consumption is considered a continuous risk factor and relative risks are parameterized to describe the change in risk for a unit decrease in salt intake across a given range. The model considers that reduction of salt consumption by 6 g/p/d was associated with a reduction of 5.8 mmHg in systolic blood pressure (SBP) [21]. In the next step differential SBP (mmHg) from salt is estimated, considering intervals of salt intake, ranging from less than 5 to over 12 g, and adjusted to the relative risks for changes in SBP for different CVD outcomes, such as coronary heart disease (CHD), stroke, hypertensive disease and rheumatic heart disease (RHD), stratified by age group and sex. At the end the model estimates for each CVD per unit of change in salt consumption.

The cost estimates related to CVDs that are attributable to excessive salt intake were calculated using the population attributable risk (PAR) by sex and age group and multiplied by the costs of CVDs to the Costa Rican national health system (such as hospitalizations, consultations, and medications). Cost data for 2018 were obtained from the CCSS.

PAR was calculated by the following formula:

PAR=100xP(RR−1)/(P(RR−1)+1)

Where:

P = The prevalence of excessive salt consumption, and

RR = The combined relative risk of sodium consumption leading to increased blood pressure, and blood pressure leading to CVD outcomes.

We used nominal costs for 2018, without any adjustment for inflation, in Costa Rican colones and s converted to U.S. dollars (USD $), at an exchange rate of USD $1 = CRC ₡600 (by December 31, 2018).

Considering the uncertainty of outcomes in the model, performing a probabilistic sensitivity analysis is recommended to incorporate the potential effects of reducing salt consumption on the risk factors for CVDs. In this paper, simulations were performed using the Monte Carlo methodology, which allows a stochastic (random) variation of parameters based on the sizes of the effects obtained from the literature. By using this technique, the model results were recalculated iteratively and uncertainty intervals of 95% (UI 95%) were generated for the median using the bootstrap percentile method. The model simulation was implemented with the Monte Carlo analysis using the specified probabilistic distributions for the model input variables (salt intake, costs, and relative risks).

The Monte Carlo simulation in the model estimated uncertainty intervals (UI) for the attributable costs [23]. Each simulation runs 10,000 times (draws) and, for each iteration, log-normal distributions are assumed for the input parameters, resulting in 95% uncertainty intervals, based on the 2.5 and 97.5th percentiles of the simulations.

All input variables and data sources for the cost-of-illness estimations are summarized in Table 1.

**Table 1:** Summary of the key model inputs and sources.

Model inputs	Value	Source
Life expectancy in women and men	82.6 (women) and 77.5 (men)	GBD IHME [21].
Rate of the economically active population (employment rate)	0.62	INEC [23]
Average income of the economically active population	USD $765	INEC [23]
Adjusted salt intake (the terms is assumed equivalent as sodium intake across population groups).	10.0±5.4 g/d/p at 2171 kcal/d	Blanco-Metzler et al. (2017) [24]. During the analyses these were the data available
Counterfactual scenario	5.0±2.65 g/d/p at 2171 kcal/d	WHO maximum recommendation of salt intake/day and daily caloric intake of the CR population according to Blanco-Metzler et al. (2017) [24].
Total, cost of consultations	USD $853,309,618.14	CCSS (S2 Appendix).
Total, cost of hospitalizations	USD $1,584,985,326.39	CCSS (S2 Appendix).
Medication costs for hypertension and CHD	USD $ 642 090.81	CCSS (S2 Appendix).

Hypertension: High Blood Pressure

CHD: Coronary Heart Disease

## Results

### Estimated total costs of hospitalization, consultation, and medications by CVD type for the CCSS due to excessive salt intake

The total annual costs of hospitalizations, consultations, and medications attributable to excessive salt intake in the population older than 15 years of age for 2018, were estimated at USD $15.1 million. The highest costs were in hospitalizations (53%), followed by consultations and medications (32 and 15%, respectively) (Table 2).

**Table 2:** Estimated total costs (thousand USD $) of cardiovascular disease hospitalizations, consultations, and medications, and losses of Gross Product by cardiovascular disease type for CCSS attributable to excessive salt intake in Costa Rica, 2018 (95% uncertainty intervals).

	Hospitalizations	Consultations	Medications	Total
Cardiovascular diseases	8,019	4,905	2,198	15,122
	(7,611–8,431)	(4,658–5,154)	(2,077–2,323)	
Coronary heart disease	3,108	2,261	221	5,589
	(2,965–3,252)	(2,157–2,366)	(210–231)	
Stroke	2,433	1,310	-	3,742
	(2,357–2,508)	(1,269–1,350)		
Hypertensive disease	1,528	823	1,977	4,328
	(1,442–1,616)	(776–870)	(1 866–2 092)	
Rheumatic heart disease	951	512	-	1,463
	(847–1,055)	(456–568)		

Among CDV total costs broken down by type for the CCSS, 37% (USD $5.6 million) were attributed to CHD, 25% to stroke (USD $3.7 million), 29% to hypertensive disease (USD $4.3 million), and 10% (USD $1.5 million) to RHD (Table 2).

### Estimated hospitalizations costs by CVD type and gender for CCSS due to excessive salt intake

Annual hospitalizations costs by type of CVD due to excessive salt intake were estimated at USD $8 million. Of these 39% (USD $3.1 million) were attributed to CHD, 30% to stroke (USD $2.4 million), 19% to hypertensive disease (USD $1.5 million) and 12% to RHD (USD $ 0.9 million) (Table 3).

**Table 3:** Estimated costs (thousand USD $) of hospitalizations by CVD type and gender for CCSS due to excessive salt intake in Costa Rica, 2018 (95% uncertainty intervals).

	Hospitalizations		
	Men	Women	Total
Cardiovascular diseases	3,867	4,153	8,019
	(3,671–4,064)	(3,940–4,367)	(7,611–8 431)
Coronary heart disease	1,702	1,406	3,108
	(1,624–1,781)	(1,342–1,471)	(2,965–3,252)
Stroke	1,126	1,306	2,433
	(1,091–1,161)	(1,266–1,347)	(2,357–2,508)
Hypertensive disease	585	943	1,528
	(552–619)	(890–997)	(1,442–1,616)
Rheumatic heart disease	453	498	951
	(404–503)	(443–552)	(847–1,055)

Total hospitalization costs were higher in women (52%) compared to men (48%) and according to the type of CVD, except for CHD, that was higher among men compared to women (Table 3).

### Estimated consultations costs by type of CVD and gender for the CCSS due to excessive salt intake

The annual consultation costs according to the type of CVD attributable to excessive salt intake were estimated at USD $4.9 million. The total costs according to gender were higher in men (54%) compared to women (46%) (Table 4). Of the total costs, 46% (USD $2.3 million) were attributed to CHD, 27% to stroke (USD $1.3 million), 17% to hypertensive diseases (USD $0.8 million), and 10% to RHD (USD $0.5 million) (Table 4).

**Table 4:** Estimated costs (thousand USD $) of consultation by CVD type and gender for the CCSS due to excessive salt intake in Costa Rica, 2018 (95% uncertainty intervals).

Diseases	Consultations		
	Men	Women	Total
Cardiovascular diseases	2,645	2 260	4 905
	(2,514–2,777)	(2,145–2,377)	(4,658–5,154)
Coronary heart disease	1,479	782	2,261
	(1 411–1 548)	(746–818)	(2,157–2,366)
Stroke	606	703	1,310
	(588–625)	(681–725)	(1,269–1,350)
Hypertensive disease	315	507	823
	(297–333)	(479–537)	(776–870)
Rheumatic heart disease	244	268	512
	(217–271)	(239–297)	(456–568)

### Estimated medications costs by CVD and gender for the CCSS due to excessive salt intake

Annual medication costs by type of CVD due to excessive salt intake were estimated at USD $2,2 million. Costs were higher among women (60%) compared to men (40%) (Table 5).

**Table 5:** Estimated costs (thousand USD $) of medications by CVD and gender for CCSS due to excessive salt intake in Costa Rica, 2018 (95% uncertainty intervals).

Diseases	Medicines		
	Men	Women	Total
Cardiovascular diseases	868	1,329	2,198
	(821–917)	(1,256–1,406)	(2,077–2,323)
Coronary heart disease	141	79	221
	(135–148)	(76–83)	(210–231)
Hypertensive disease	727	1,250	1,977
	(686–769)	(1,180–1,323)	(1,866–2,092)

Of the total costs, 90% (USD $2.0 million) are attributed to hypertensive diseases and the remaining 10% to CHD (USD $0.2 million) (Table 5). Due to the unavailability of CCSS medication expenditure data for all CVDs, it was not possible to estimate medication costs for stroke and RHD (Table 5).

### Estimated costs of productivity losses by CVD type and gender for CCSS due to excess sodium intake

The annual productivity costs of losses by type of CVD to the Costa Rican Gross Domestic Product (GDP), attributable to excessive salt intake, were estimated at USD $6.8 million, resultant of 2,275 YPLL due to disability due to CVD in the economically productive population. The costs were higher among women (51%) compared to men (49%). Of the total costs, 60% (USD $4.1 million) were attributed to CHD and 40% to stroke (USD $2.8 million) (Table 6).

**Table 6:** Estimated costs (thousand USD $) of productivity losses by CVD type and gender for CCSS due to excessive salt intake in Costa Rica, 2018.

CDV	Productivity losses		
	Men	Women	Total
Cardiovascular diseases	3,340	3,490	6,830
	(3,207–3,473)	(3,350–3,632)	(6,557–7,105)
Coronary heart disease	1,932	2,141	4,073
	(1,843–2,021)	(2,043–2,240)	(3,886–4,262)
Stroke	1,408	1,349	2,757
	(1,364–1,452)	(1,307–1,391)	(2,671–2,843)

## Discussion

This research showed that the costs of CVD care attributable to excessive salt intake for the CCSS and the GDP losses due to premature deaths from CVD represent important economic burdens to the country that could be saved by reducing salt intake by the population. In total, the direct costs of treatment of CVDs attributable to excessive salt intake to the Costa Rican national health system are estimated at USD $15.1 million per year, considering data from 2018. Additionally, the indirect costs related to the premature CVD deaths attributable to excessive salt intake represent a significant economic burden to the country in terms of losses of productivity (USD $6.8 million/year).

This type of study is one of the few carried out in Costa Rica and details the estimation of direct and indirect costs of excessive salt intake associated with CVDs. There are limited national data on the evaluation of costs of risk factors associated with NCDs, especially considering dietary risk factors. The most recent official data corresponded to that indicated by the CCSS on hypertension costs in 2012 represented 3.47% of social security spending [10].

In this regard, in 2015, another study estimated the economic benefit and viability for the CCSS of the complete implementation of the National Plan for the Reduction of the Consumption of Salt and Sodium in the Population of Costa Rica over a period of 10. With full implementation of the Plan, reducing the maximum salt intake to 5 g/p/d, the CCSS would save USD $88 million in 10 years, i.e., approximately USD $9 million for year (CI: USD $43,527,720.87 and USD$187,045,390.32) [11] in hospitalizations, consultations, and medications, which represents less than the costs estimated in this study. However, both investigations have different objectives and methodologies, which may have influenced their results. However, the expenses incurred by the CCSS may be much higher, together with other costs not quantified in these studies, including primary health care costs and treatments for other CVDs and NCDs related to hypertension.

Additionally, according to the 2015 investigation, if the prevalence of hypertension was reduced from 31% to 26%, the projected costs of hypertension to the CCSS would be reduced from USD $557 million to USD $ 469, in 10 years. This indicates that the savings associated with salt reduction to the CCSS may be greater, due to unquantified costs, longer evaluation period, effects of the decrease in comorbidities associated with hypertension, and cost of opportunity [11].

The management of hypertension and associated diseases conservatively consumed around 10% of global spending on medical care, because if the welfare losses due to premature death were added, the costs could be 20 times higher [1]. Therefore, PAHO/WHO states that population interventions to salt reduction could considerably reduce the burden of NCDs to health systems, in addition to saving millions of lives each year, and represent one of the most effective and cost-effective strategies for reducing the burden of NCDs [2].

Therefore, our findings are consistent with the magnitude burden estimated by other studies in Costa Rica, yet are different from various international publications, considering the trends observed on the significant economic benefits of interventions to reduce salt consumption at the population level [1]. This finding indicates that for low and middle income countries like Costa Rica, results must be contextualized according to the population, economic, epidemiological characteristics, and the model of the social security system; which are different from the scenarios in high income nations [25].

In the present study, the highest costs were from hospitalizations (53%), followed by consultations and medications (32% and 15%, respectively). Current literature indicates that acute coronary syndromes (“heart attacks”) and strokes are typically treated in hospitals and require advanced medical, diagnostic, and sometimes surgical skills; for which it seems to coincide with the results obtained in the present investigation [26].

Likewise, it is recognized that NCDs are the main causes of outpatient consultations, especially in the case of hypertension and CVD (about 1 in 4 people) [27].

Unfortunately, there is little research data on the cost-effectiveness of the different therapeutic options in Latin American countries, which limits the ability to compare the results with respect to the cost of drugs. However, studies on secondary prevention and long-term care of cardiovascular disorders suggest that certain pharmacological therapeutic regimens and certain technologies can be cost-effective for both situations [27].

To this end, evidence has shown in the UK estimated that achieving a dietary salt intake of less than 6 g/d could potentially reduce the need for antihypertensive drugs by up to 30%, and a 10% reduction in salt intake in that country since 2000 by gradual and sustained industry efforts to reduce salt in certain food products and other strategies has produced a £1.5bn cost saving benefits [28].

When analyzing the total costs by type of disease, most of them are attributed to CHD (37%), followed by hypertension (29%), stroke (25%) and, finally, 10% to RHD.

A recent systematic review of the economic burden of CVD and hypertension in low- and middle-income countries found the costs of CHD and stroke were generally higher and heterogeneous [29]. In Costa Rica (2016–2017), the average cost to care for a patient in cardiac rehabilitation centers was Int $867 (Purchasing Price Parity, expressed as International Dollars or Int $) and the total costs rehabilitation programs per patient was Int $300 [30]. Similarly, in Colombia, the costs of hypertension represent a significant percentage of expenses from medications and referrals to other specialties or treatments [31].

According to other studies, stroke care represents the highest cost among CVDs [3, 4] and the economic costs of post-stroke care are enormous (to date, approximately 34% of the world’s total health spending) [3, 4].

The evidence indicates that the response of the health system to provide sufficient treatment and health technology is limited for these diseases [32, 33]. This is explained in part by accelerated demographic (e.g., longer life expectancy) and epidemiological changes, in a context of slow preparation for investment aimed at controlling NCDs-DR [34].

Considering the indirect costs of salt intake, this study estimated that losses of 2,275 YPLL were attributable to excessive salt intake and the corresponding losses of productivity due to premature deaths account for a total annual loss of 1% of the country’s GDP. In 2013, the economic burden of hypertension of GDP ranged between 2.5% and 8% of GDP in developing countries, while in developed countries it was between 5% and 15% [35]. Similarly, the high prevalence of diet related NCDs reduces the GDP of countries and their potential productivity because of work absenteeism associated with disability, illness and premature death. In 2017, public spending on health in the countries of the region varied between 2 and 7% of GDP; even in six countries it was equal to or less than 5% [36].

Economic evaluations of strategies such as the reduction of salt consumption can benefit national food policies to estimate the costs of inadequate diets in low- and middle-income countries, such as Costa Rica, as has happened with the evaluation of anti-smoking strategies [12]. In addition, these studies complement the estimated morbidity and mortality impacts, as improving treatment and prevention strategies and, when necessary, targeted policies for specific population groups, with different needs and realities [12].

The evidence indicates that policy costs may represent a potential barrier to the implementation of recommended strategies to reduce salt intake because many countries have limited resources for health interventions, which requires a careful evaluation of their profitability. The profitability of such efforts worldwide is uncertain, since there are few studies in this regard and focused mainly in high-income countries, so results generally not comparable [37, 38]. Similarly, the existing evidence on the economic burden of CVD in low- and middle-income countries does not appear to be aligned with political priorities in terms of volume of research, pathologies studied, and methodological quality. In the future, country-level studies with appropriate sample sizes are needed, with adequate incorporation of indirect costs to replace retrospective, institutional, and small-scale cost studies, and with high-quality data, to provide decision makers and program developers a stronger economic case for implementing or not implementing the interventions [29, 39].

Reducing salt intake at the population level is one of the most cost-effective strategies to reduce the risk of CVD´s and premature deaths [40, 41]. In order to support national salt reduction policies, WHO developed the SHAKE package as a tool for developing countries to design, implement and monitor evidence-based salt reduction strategies [2].

Among salt reduction strategies, multicomponent interventions are more effective than isolated interventions [42]. These interventions include salt targets in processed foods, mandatory nutrition facts, front of package warning labeling, fiscal measures, regulation of the marketing and promotion of unhealthy foods and education and social marketing programs or campaigns. For example, in the United Kingdom and Finland, coordinated salt reduction strategies produced significant health outcomes, by reducing blood pressure at the population level and the burden of CVD and even improving the life expectancy in 5 to 6 years [43–45].

Therefore, studies on population modeling of disease burden can play an important role in informing policy makers about the efficacy of a salt reduction strategy for the entire population [46]. These studies allow us to understand the economic costs of a particular disease, since they identify several components or complications related to it, which could have been avoided if it were not present [27]. This is incredibly relevant in a public health system with limited resources.

The strengths of this study include the use of nationally representative data from Costa Rica concerning the deaths and costs to the national health system and official data from national surveys, labor force characteristics and national life table estimates. These macrosimulation approaches are based on comparative risk assessment models, such as the Global Burden of Disease Study, so intra- and inter-country and regional comparisons are possible under similar conditions relating to the relative risks associated with cardiovascular diseases.

Modeling studies using probabilistic approaches represent an estimate of the magnitude of the impact in different policy or counterfactual scenarios and must be interpreted as so. The uncertainty intervals of the results incorporate the uncertainty of many of its inputs, including the uncertainty of risk factor exposure (as the variance in sodium intake) and relative risks from meta-analyses, through the sensitivity analysis.

The macrosimulation approaches for cost-of-illness estimates have several limitations. Firstly, the model is subject to the limitations of the data sources used to determine the inputs of the model, although it was prioritized to use the best available and most representative data, including the INEC, the CCSS and robust meta-analyses [21, 22].

Additionally, comparative risk assessment models do not incorporate the effect of time lag between changes in exposures and disease outcomes. Also, the relative risk estimates used to parameterize the models are based on published meta-analyses from other settings, mainly high-income countries and these macrosimulations do not account for all sources of data heterogeneity in populations.

As a result, the cost estimates are likely underestimated, considering the multiple direct and indirect costs of CVDs. The analyses also do not account for health insurance and out-of-pocket costs, as well as the costs of hypertension and CVDs to primary health care.

Finally, assumptions were made to make the model usable and manageable, such as assuming equivalent sodium intake across population groups.

## Conclusions

NCDs caused by excessive salt intake represent important economic losses for Costa Rica, not only in terms of direct health costs (consultations, hospitalizations, and medications), but also indirect costs due to the losses of productivity due to premature CVD deaths and, therefore, losses in the GDP and the undermining the development of Costa Rica. This is the first study of its kind in the nation and has paramount importance to policymakers, providing reliable and useful estimates of CVD care costs attributable to excessive salt intake for CCSS, GDP losses and YPLL for CVD.

Considering the results, it is recommended that Costa Rica should consider setting a national surveillance, monitoring and evaluation program for reducing the intake of salt and other critical nutrients identified as dietary risk factors for NCDs. In addition, policymakers should consider the adoption a strategy that combines multiple interventions (food reformulation, mandatory nutrition facts, front of package warning labeling, fiscal measures, regulation of the marketing and promotion of foods with an excess content of nutrients associated with NCDs-DR, and education and marketing programs or campaigns for the reduction of salt consumption in the population). These strategies would contribute to generating a healthier food environment in the country, to facilitate healthy decision-making and thus reduce the epidemiological and economic burden of the high consumption of salt and other nutrients of interest in public health.

## Supporting information

S1 Appendix (XLSX)

S2 Appendix (XLSX)
